# A Ten year review of alcohol use and major trauma in a Canadian province: still a major problem

**DOI:** 10.1186/s13032-016-0033-x

**Published:** 2016-01-21

**Authors:** Jessica McKee, Sandy L. Widder, J. Damian Paton-Gay, Andrew W. Kirkpatrick, Paul Engels

**Affiliations:** Alberta Centre for Injury Control and Research, School of Public Health, University of Alberta, Edmonton, AB Canada; Department of Surgery and Critical Care, University of Alberta, Edmonton, AB Canada; Department of Surgery and Critical Care Medicine, University of Calgary, Edmonton, AB Canada; Departments of Surgery and Critical Care Medicine, McMaster University, Hamilton, ON Canada

**Keywords:** Trauma, Alcohol, Injury prevention

## Abstract

**Background:**

Alcohol plays a significant role in major traumatic injuries. While the role of alcohol in motor vehicle trauma (MVT) is well described, its role and approaches to prevention in other injury mechanisms is less defined.

**Methods:**

A 10 year retrospective examination of Alberta Trauma Registry (ATR) data was conducted on all major trauma patients (age ≥ 9 and ISS ≥ 12) from 2001–2010. The role and prevalence of alcohol is examined.

**Results:**

Of 22,457 patients included in our study, only 60 %(*n* = 13,552) were screened for alcohol use. Of those screened, 38 %(*n* = 5,170) tested positive for alcohol with a mean blood alcohol concentration (BAC) of 39.4 ± 21.1 mmol/L. Of the positive screening tests, 82.3 % had BAC levels greater than the common legal driving limit of 17.4 mmol/L (0.08 %). Testing positive was associated with male gender (*p* < 0.001) and younger age (*p* < 0.001). The rate of positive alcohol use in major trauma increased from 20.3 % in 2001 to 24.3 % in 2010, corresponding with a screening rate increase from 51.3 % to 61.2 % over the same period. Railway incidents have the highest rate of alcohol involvement (65 %), followed by undetermined-if-accidental/self-inflicted (53.5 %) and assault (49 %); motor vehicle traffic (MVT) incidents had a frequency of 25.4 %.

**Conclusions:**

The prevalence of alcohol use in major trauma appears to be increasing in Alberta but the true extent is still underappreciated. Furthermore, the role of alcohol in non-MVT injuries is significant and deserves further attention. The vast majority of patients involved in alcohol-related trauma are legally intoxicated. Alcohol use continues to be a substantial contributor to major trauma in Alberta, and represents an important opportunity to reduce preventable injuries.

## Background

Alcohol abuse is a major contributing factor in many types of injuries. Globally, alcohol use contributes to 1 in 25 deaths and is responsible for 1 in 20 disability-adjusted life years [1]. Its role in motor-vehicle collisions (MVC) has long been recognized [2] and has more recently been reported to be involved in 4 of every 10 fatal motor vehicle collisions [3]. Although knowledge of the perils and criminality of “drunk-driving” have been made socially prevalent, partly through the contributions of organization such as Mothers Against Drunk Driving (MADD) [4], government programs such as Reduce Impaired Driving Everywhere (RIDE) [5] and Checkstop [6, 7], driving while inebriated continues to be a significant cause of preventable injury and death. In 2009, it is estimated that over 1,000 people were killed and over 60,000 were injured in alcohol-related collisions in Canada alone [4].

While the issues surrounding “drinking and driving” tend to be well-recognized and publicized, the contribution of alcohol to other causes of traumatic injury is, in general, under-recognized and underappreciated. Although alcohol is widely recognized to impair judgement and motor function, it also provokes hazardous activity by unmasking aggression and promoting impulsiveness [8]. In addition, alcohol has many physiologic effects that negatively alter the body’s response to injury [9] and complicates the ability of healthcare providers to evaluate and care for such patients [10]. Studies published in the early 1990s demonstrated that approximately 40 % of patients admitted to trauma centres had alcohol use as the precipitating factor leading to acute injury [11], with a significant prevalence of substance abuse disorders also reported in this population [12]. More recent studies have demonstrated alcohol use to be a significant risk factor for various injury types ranging from falls to interpersonal violence to recreational vehicle use [13–15].

Although the contribution of alcohol to traumatic injury has long been known, the opportunities to systematically identify these individuals and offer interventions to decrease recidivism have only recently been recognized [16] and proven effective [17–19]. Indeed, screening of trauma patients for blood alcohol concentration (BAC) was rare in the 1990s. The recent recommendation for mandatory blood alcohol screening of trauma patients in the USA [20] and Canada [21] seeks to identify and target those involved in alcohol-related trauma, in an attempt to decrease their recidivism [16].

In our study, we sought to perform a comprehensive review of all alcohol-related trauma in our province over the previous decade in order to determine the contribution of alcohol in it’s entirety to the total burden of injury, and to better identify opportunities for both primary and secondary injury prevention.

## Methods

### Data Collection

This injury surveillance effort is a retrospective review of the role and prevalence of alcohol in major trauma injury. Data was obtained from the Alberta Trauma Registry (ATR), which is housed at the Alberta Centre for Injury Control and Research (ACICR). The ATR prospectively collects information on all major trauma patients (Injury Severity Score [ISS] ≥ 12) treated at level 1, 2, and 3 trauma centres in Alberta. We included all patients that were entered into the ATR from January 1, 2001 – December 31, 2010 and whose age ≥ 9. This age cut off was selected as this is the cut off age used for the National Trauma Registry (NTR) [22].

### Research Design

Data obtained from the ATR included age; sex; type of injury; death at scene; admission status; total hospital length of stay (LOS); intensive care unit (ICU) LOS; ISS; blood alcohol concentration (BAC); mechanism of injury; date of injury and discharge status.

### Statistical Analysis

Statistical analysis was completed in SPSS software, version 19 (IBM Corporation, Armonk, New York). In cases where multiple BAC readings were recorded per patient (due to hospital transfer), the highest BAC level recorded was used for the statistical calculation. We compared clinical and demographic variables between patients that had BAC screening and no screening. Of those screened, we also compared patients with a positive (any alcohol detected) versus negative (<2 mmol/L) BAC screen. All reported BAC levels (negative or positive) in the ATR were considered to have been drawn as a screening test. We performed Student t tests to compare age and BAC. The Mann-Whitey U test was used to compare total LOS, ICU LOS, ISS and the X^2^ test to compare sex, type of injury, death at scene, direct admission and discharge status. Positive alcohol levels were also stratified according to Dubowski’s [23] stages of alcohol intoxication.

## Results

There were 22,457 patients that met inclusion criteria for the study. Of the 22,457 patients included 16,715 (74.4 %) were male and 5742 (25.6 %) were female, ranging in age from 9 to 102 (median 41.0 [interquartile range; IQR 34.0]) years. Eight hundred sixty-five (3.9 %) patients had a death on scene (2,317 patients were missing death on scene information), 855 (3.8 %) were a direct admission (13 patients were missing admission status), 20,886 (93.0 %) patients sustained a blunt injury, 1,205 (5.4 %) sustained a penetrating injury and 366 (1.6 %) sustain another form of injury. The study sample has an ICU LOS median 0.0 (IQR 2) days, hospital LOS median 7.0 (IQR 12) days and ISS median 21 (IQR 10; 3 patients were missing an ISS score).

Table 1 displays the comparison between BAC screened and not screened groups. Screening for BAC levels is more likely in younger, male patients. Penetrating trauma, associated death on scene, emergency admission to hospital versus direct admission and more severely injured patients (as determined by higher LOS and ISS) were also significantly more likely to elicit BAC screening.

**Table 1:** Characteristics of patients screened and not screened for alcohol

Characteristic		Screened	Not Screened	*p*-value
Sex	Male:Female Ratio	10492:3060	6223:2682	<0.001^a^
Injury Mechanism	Blunt:Penetrating Ratio	12502:887	8384:318	<0.001^a^
Associated Death at Scene	No Death:Death Ratio	11712:636	7563:229	<0.001^a^
Admission Status	Not Direct:Direct Ratio	13231:226	8268:629	<0.001^a^
Age	Years^d^	37.0 (27)	49.0 (46)	<0.001^b^
Total LOS	Days^d^	8.0 (12.0)	6.0 (10.0)	<0.001^c^
Total ICU LOS	Days^d^	8.0 (12.0)	6.0 (10.0)	<0.001^c^
ISS	Score^d^	22.0 (13.0)	18.0 (9.0)	<0.001^c^

^a^by chi-squared analysis

^b^by Student’s t-test

^c^by Mann–Whitney U test

^d^values reported as median (IQR)

Of those screened (*n* = 13,552; 60.3 %), Table 2 displays the comparison between screened BAC negative and positive groups. A positive BAC level is more likely in younger, male patients. Penetrating mechanism and having no associated death on scene, were also significantly more likely to be positive for alcohol use.

**Table 2:** Characteristics of patients who screened positive versus negative for alcohol

Characteristic		Alcohol Positive	Alcohol Negative	*p*-value
Sex	Male:Female Ratio	4345:825	6147:2235	<0.001^a^
Injury Mechanism	Blunt:Penetrating Ratio	4594:517	7908:370	<0.001^a^
Associated Death at Scene	No Death:Death Ratio	4493:191	7219:445	<0.001^a^
Admission Status	Not Direct:Direct Ratio	5089:78	8232:148	0.270^a^
Age	Years^d^	33.0 (22.0)	40.0 (31.0)	<0.001^b^
Total LOS	Days^d^	7.0 (12.0)	8.0 (13.0)	<0.001^c^
Total ICU LOS	Days^d^	3.74 (9.8)	3.35 (11.3)	<0.001^c^
ISS	Score^d^	22.0 (13.0)	22.0 (13.0)	0.056^c^
BAC	mmol/L^d^	38.0 (31.0)	0.0 (0.0)	<0.001^b^

^a^by chi-squared analysis

^b^by Student’s t-test

^c^by Mann–Whitney U test

^d^values reported as median (IQR)

**Fig. 1:**
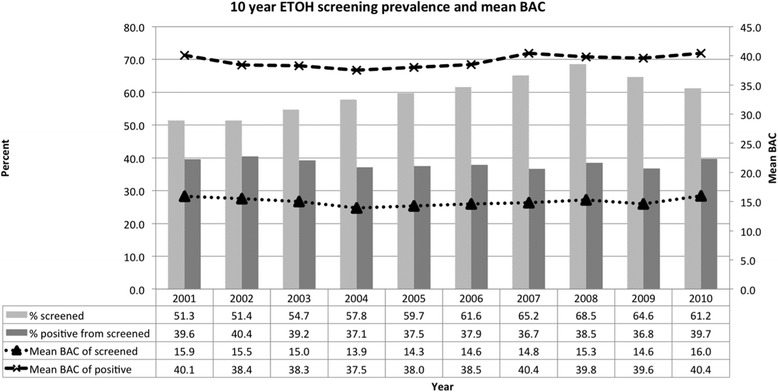
10 year BAC screening prevalence rate and mean BAC (mmol/L)

Figure 1 illustrates the screening prevalence rate, positive screening rate, mean BAC of those screened, and mean BAC of those screening positive for the 10 year study period. The average screening rate rose from 51.3 % in 2001 to a high of 68.5 % in 2008 and has since fallen. The positivity rate of those screened has remained fairly constant at approximately 38 %.

**Fig. 2:**
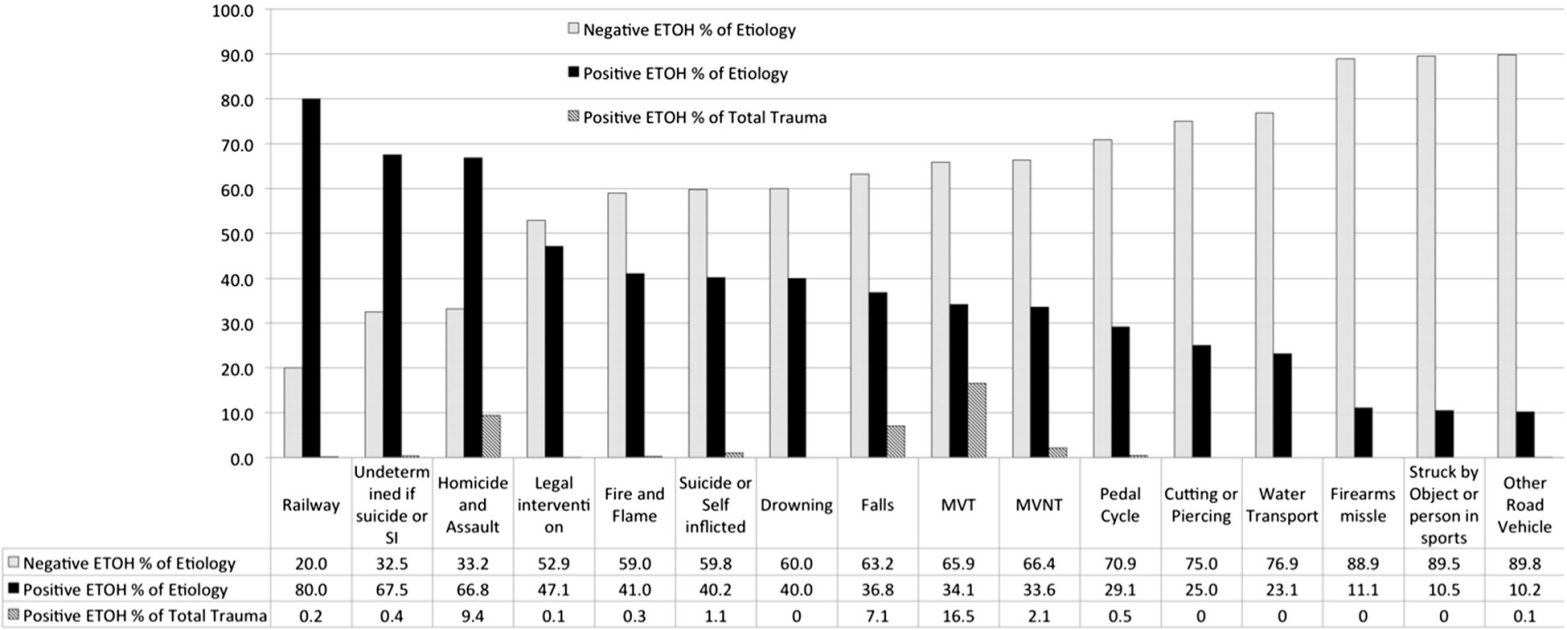
Rate of positive and negative BAC screening by/within mechanism of injury

Mechanism of injury related to positive BAC rates is displayed in Fig. 2. Railway incidents had the highest percentage of positive alcohol screens followed by “undetermined if suicide or self inflicted”, then “homicide and assault”. Motor vehicle trauma (MVT) is ranked 9^th^ with 34.1 % of such patients testing positive for alcohol. When accounting for total trauma volumes, motor vehicle traffic had the highest incidence of alcohol use (16.5 %), followed by homicide/assault (9.4 %), and falls (7.1 %).

**Fig. 3:**
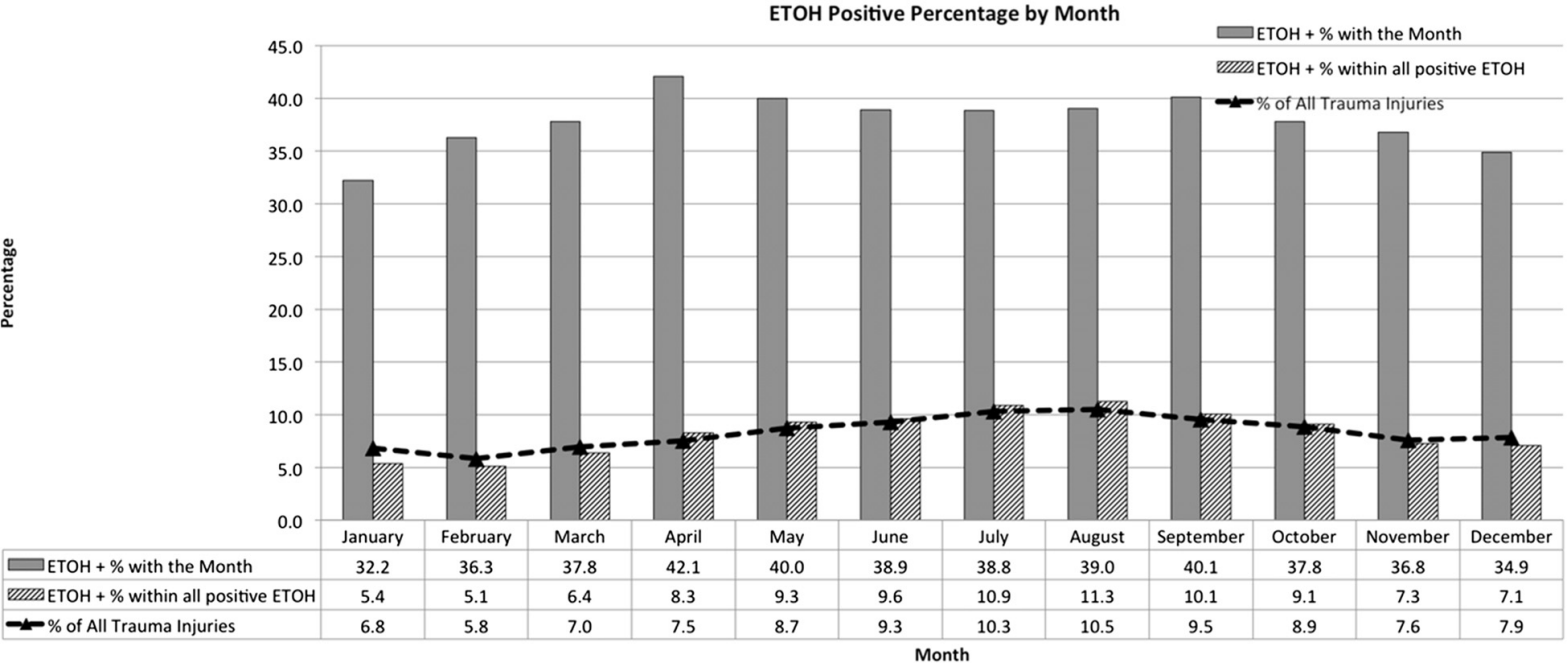
BAC screening rate and injury rate by month

**Fig. 4:**
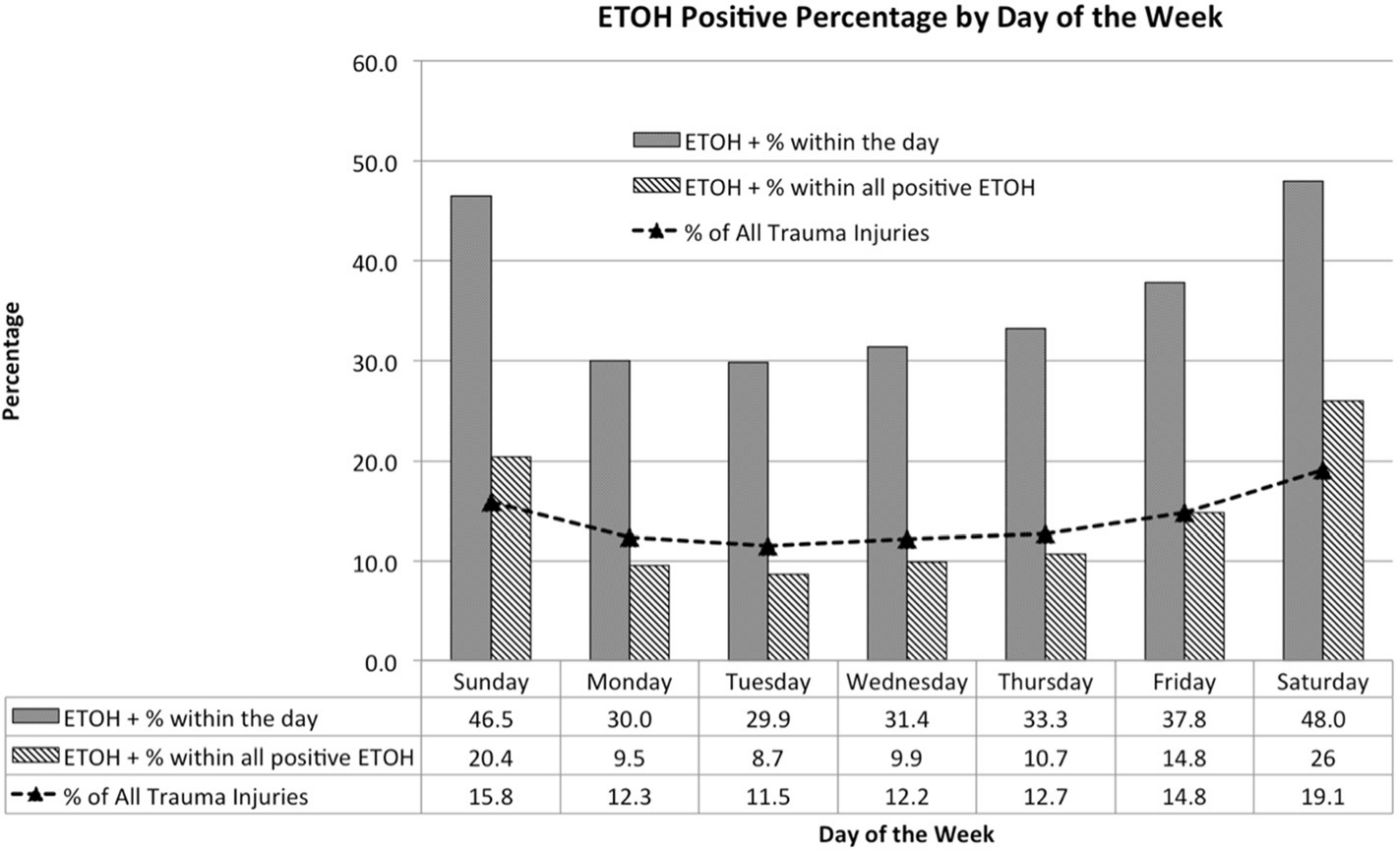
BAC screening rate and injury rate by day of the week

The relationships between positive BAC levels according to month of the year (Fig. 3) and day of the week (Fig. 4) are displayed. The greatest percent of BAC positive injuries occured during July and August. The volume of trauma and the proportion that is BAC positive both peaked on the weekend.

The distribution of ethanol positive trauma cases (*n* = 5170), in a system of overlapping ethanol levels, according to Dubowski’s [23] stages of acute alcohol influence/intoxication, is displayed in Table 3. The mean ethanol levels of those who screened positive was 39.4 ± 21.1 mmol/L (ranging from 2.00 mmol/L – 134 mmol/L ). When compared to common legal BAC limits for operating a motor vehicle, 90.1 % of BAC positive patients had a BAC ≥ 11 mmol/L (equivalent to 0.05 % or 50 milligrams of alcohol for every 100 milliliters of blood) and 82.3 % were ≥ 18 mmol/L (equivalent to 0.08 %). Furthermore, 4.8 % had a BAC ≥ 65.2 mmol/L, which can be a lethal level.

**Table 3:** Distribution of alcohol levels in all trauma patients

BAC (mmol/L)	Stage	Symptoms	Alcohol + N = 5170	Alcohol Level mean ± SD (mmol/L)
2.00 – 10.85	Subclinical	Behavior Normal	488 (9.4 %)	6.17 ± 2.4
6.51 – 26.04	Euphoria	Difficulty concentrating	1268 (24.5 %)	16.98 ± 5.7
		Talkative		
		Lowered inhibition		
		Brighter color in face		
		Fine motor skills are lacking		
19.53 – 54.25	Excitement	Senses are dulled	2863 (55.4 %)	37.25 ± 9.7
		Poor coordination		
		Drowsy		
		Beginnings of erratic behavior		
		Slow reaction time		
		Impaired judgement		
39.06 – 65.10	Confusion	Exaggerated emotions	1995 (38.6 %)	50.81 ± 7.4
		Difficulty walking		
		Blurred vision		
		Slurred speech		
		Pain in dulled		
58.59 – 86.8	Stupor	Cannot stand or walk	1152 (22.3 %)	65.14 ± 8.2
		Vomiting		
		Unconsciousness is possible		
		Decreased response to stimuli		
		Apathetic		
75.95 – 108.5	Coma	Unconscious	239 (4.6 %)	85.47 ± 7.5
		Low body temperature		
		Possible death		
		Shallow breathing		
		Slow pulse		
>97.6	Death	Death due to respiratory failure	27 (0.5 %)	106.38 ± 8.3

BAC, Blood Alcohol Concentration

Alcohol+, positive screening for alcohol

SD, Standard Deviation

## Discussion

In line with recent recommendations [20, 21] and initiatives [17, 18, 24–26] to increase screening for alcohol use in trauma patients, screening rates have improved over the past decade in Alberta rising from 51 % to 61 %, with a peak screening rate of 68.5 % in 2008. Despite known challenges in screening this patient demographic [19, 27, 28], many strategies exist [17, 18, 24, 25] with a screening rate between 72 % and almost 100 % reported by some centres [29–31]. While Alberta has made progress in improving screening rates, the opportunity for intervention in alcohol-related trauma is dependent on *universal* screening [20, 32]; without such screening, intervention and prevention opportunities will be lost. Although recommended by national guidelines [21], the successful implementation of local alcohol SBIRT (Screening, Brief Intervention, and Referral to Treatment) programs requires necessary resources and personnel that are not currently provided; such dedicated funding represents an opportunity to improve alcohol screening.

Research has demonstrated that alcohol problems are treatable and interventions can be successful. Indeed, in a Cochrane Database Review published in 2004, the authors concluded “[i]nterventions for problem drinking appear to reduce injuries and their antecedents (e.g. falls, motor vehicle crashes, suicide attempts)” [32]. A recent study by Gentilello and colleagues demonstrated alcohol interventions to be cost effective as a consequence of decreasing future traumatic events and health-care costs [20]. In addition, alcohol-related traumatic injury affects not just young adults, but the pediatric and geriatric populations as well. Thus, any screening and intervention programs should also include these important demographics [33, 34].

Of the patients who received ethanol screening in Alberta, almost 40 % were positive each year throughout the decade of study. This value is consistent with previous studies [12, 31] and demonstrates the ongoing linkage between ethanol use and major trauma. It should be noted that the positive screening rate has not changed, despite an increase in the overall alcohol screening rate; thus more patients continue to be identified via screening, and suggests that the incidence of alcohol use in major traumas may be much higher than currently or previously estimated. It is clear that alcohol use has been a consistent and prevalent factor in injury occurrence over the last decade and the role of alcohol in major trauma remains significantly underestimated.

Although the relation between motor vehicle trauma (MVT) and alcohol is well publicized, societal appreciation of the role of alcohol in other types of major trauma is under-appreciated. Despite having significant resources dedicated to the problem of drinking and driving, the rates of alcohol use in collisions both fatal and involving injury have not declined in Alberta [35]. This may relate to a larger societal problem, in which simply targeting drivers is inadequate. Our study demonstrated a much wider range of injury prone behaviours including the mechanisms of “railway”, “undetermined if suicide of self-inflicted”, “homicide&assault”, “legal intervention”, “fire and flame”, “suicide or self-inflicted”, “drowning”, and “falls” that *all* ranked above MVT in terms of rate of BAC positivity. This data unmasks the substantive role of alcohol in an extensive array of trauma mechanisms, and identifies very important opportunities for intervention and potential public education strategies.

Not only is the rate of alcohol use in trauma significant, but the alcohol levels in trauma patients are substantial. When stratified according to Dubowski’s [23] stages of acute alcoholic intoxication/influence, we see that >50 % of all trauma patients with a positive screen were at least in the ‘Excitement’ stage, where impaired judgment is notably exhibited. Indeed, >20 % had an alcohol level that generally precludes standing or walking. If viewed from the perspective of the common legal limits to operate a motor vehicle—levels deemed to have acceptable reaction times, judgment, concentration, and coordination to safely pilot a multi-thousand pound piece of metal at highway speeds on public roads—then <10 % of those that screened positive had alcohol levels that would allow them to legally drive! Directly comparative data to other populations is difficult to obtain. Savola et al. [36] reported positive alcohol levels in 57 % of trauma patients with 86 % having levels >22 mmol/L, although mean ISS of their population was 4; Yaghoubian et al. [37] reported a positive BAC rate of only 8 %, with a median BAC of 56.3 mmol/L but the mean ISS was only 8; Swearingen et al. [38] reported a positive BAC screening rate of 34 %, with a mean level in those positive of 40.2 mmol/L although the mean ISS was 7. Our study represents a much more severely injured cohort, with the mean ISS being 21.

Although the optimal method of tackling the public health issue of alcohol and trauma is unknown, recent research in this area is uncovering potentially successful strategies. Increasing the hours of alcohol sales [39] and number of days of alcohol sales [40] has been demonstrated to increase alcohol-related harms (including alcohol-related crashes, unintentional or intentional injuries, and violent crimes). Extended hours of sale and consumption of alcohol were also found to increase the risk of homicides in a study from Columbia [41]. Thus, restricting the period where alcohol can be purchased and consumed could be expected to decrease alcohol-related harms. Decreasing the legal BAC driving limit has also been shown to decrease motor vehicle injuries and fatalities [42]. Recently, the Canadian province of British Columbia introduced laws that effectively exchanged the existing more serious but rarely applied punishment for drunk driving laws with ones that increased the certainty of apprehension albeit with lesser penalties. Analysis of the effect of these laws demonstrated significant declines in alcohol-related collisions including those with fatalities, injuries, and property damage [43]. Aside from restricting alcohol consumption, novel community programs such as the Canadian PARTY (Prevent Alcohol and Risk-related Trauma in Youth) program has demonstrated success in decreasing the incidence of traumatic injuries [44]. Indeed, a recent publication from the US Task Force on Community Preventive Services contains numerous recommended interventions to reduce alcohol-related harms, ranging from increasing alcohol taxes to mass media campaigns to school-based programs [45].

Trauma surgeons have struggled for decades to provide “optimal care” to trauma patients; however, mending their broken bones and patching up their internal organs only to have them return to the streets and highways without treatment of the underlying substance abuse disorder enables patients to continue behavior that causes injury to themselves or other people [28]. In the 21st century, the provision of “optimal care” must include injury prevention efforts. As trauma care has improved and matured, the opportunity to save lives and prevent disability has shifted from the Trauma System to the domain of public policy. Recent analyses of traumatic deaths have demonstrated that in many developed-world mature trauma systems, the lives that can be saved and the disabilities that can be prevented are by and large being so [46–48]. Although further improvements in trauma care and trauma systems will continue to be sought, efforts to address the *prevention* of traumatic injuries are projected to have a far greater impact on the overall health of a population.

Although our study only examines the relationship of alcohol with traumatic injuries, the exclusive or concomitant use of other drugs is significant in such patients and has been reported to occur in up to 27 % [49]. Furthermore, the types of drugs used is also concerning. In a United States study of alcohol-screen positive trauma patients, 15 % reported using an illicit drug that was *not* marijuana within the last 12 months [50]. The impact of alcohol and intoxicant use extends beyond the immediate injuries and includes not only patient-specific injuries, trauma recidivism, and chronic disease from their use, but has significant effects upon the direct and indirect victims (eg. friends, family, bystanders) of these traumas (eg. MVCs, interpersonal violence, etc.). Unfortunately, perpetrators of substance-related trauma do not always face consistent legal repercussions with charges of DUI(driving under the influence)/DWI(driving while intoxicated) reported in only 18 % of cases in the United States versus 85 % in Sweden [51]. The significant rate of intimate partner violence among trauma victims has also recently been recognized [52]. Alcohol abuse, substance abuse, and intimate partner abuse all demonstrate exceptional prevalence amongst trauma patients and all represent opportunities where a combined and comprehensive screening and intervention program could meaningfully contribute to improved patient- and population-based outcomes.

Our study is a retrospective analysis of a prospectively collected database and has all the inherent limitations therein. The study population included persons ≥9 years old, which may have lowered the overall screening rate as pediatric trauma patients are seldom screened for alcohol use. Screening for serum and urinary drug levels is not commonly performed in Alberta and this data is not captured in the ATR. There was missing data regarding the death on scene and admission status parameters, although given the number of patients this is unlikely to significantly impact the analysis. While screening rates appear to dip from 2008–2010, the interpretation of this is confounded by the introduction of new trauma programs during this period; thus this dip may represent under-screening in new trauma programs rather than a drop in screening rates at existing trauma programs. Although SBIRT programs for alcohol-positive patients are in effect at some centres, the ATR did not contain information on whether this service was provided or not, and thus our study cannot determine the number of alcohol screening positive patients that received an intervention. While our study focuses on Alberta, the problems of alcohol-related trauma in the developed world are ubiquitous. Indeed, the results from our study should stimulate others to examine the role alcohol is playing in traumatic injuries in their local jurisdictions and across their country.

## Conclusions

Despite increased screening rates, alcohol use continues to be widely prevalent in major trauma in Alberta. Alcohol has been shown to play a major role in not only MVT incidents but in many non-MVT mechanisms; this represents an underappreciated public health issue. As a significant cause of preventable injury, both MVT and non-MVT major trauma in Alberta continues to be heavily influenced by excessive alcohol consumption and deserves more widely focused attention if we are serious about improving the health of our populations.

## Abbreviations

ACICR: Alberta Centre for Injury Control and Research
ATR: Alberta Trauma Registry
BAC: Blood Alcohol Concentration
ICU: Intensive Care Unit
ISS: Injury Severity Score
LOS: Length Of Stay
MADD: Mothers Against Drunk Driving
MVC: Motor Vehicle Collision
MVT: Motor Vehicle Trauma
NTR: National Trauma Registry
PARTY: Prevent Alcohol and Risk-related Trauma in Youth
RIDE: Reduce Impaired Driving Everywhere
SBIRT: Screening, Brief Intervention, and Referral to Treatment
